# Xylosyltransferase I mediates the synthesis of proteoglycans with long glycosaminoglycan chains and controls chondrocyte hypertrophy and collagen fibers organization of in the growth plate

**DOI:** 10.1038/s41419-023-05875-0

**Published:** 2023-06-09

**Authors:** Mahdia Taieb, Dima Ghannoum, Lydia Barré, Mohamed Ouzzine

**Affiliations:** UMR 7365 CNRS-University of Lorraine, Biopôle, Faculty of Medicine, BP 20199, 54505 Vandoeuvre-lès-Nancy, CEDEX, France

**Keywords:** Glycobiology, Cell biology

## Abstract

Genetic mutations in the *Xylt1* gene are associated with Desbuquois dysplasia type II syndrome characterized by sever prenatal and postnatal short stature. However, the specific role of XylT-I in the growth plate is not completely understood. Here, we show that XylT-I is expressed and critical for the synthesis of proteoglycans in resting and proliferative but not in hypertrophic chondrocytes in the growth plate. We found that loss of XylT-I induces hypertrophic phenotype-like of chondrocytes associated with reduced interterritorial matrix. Mechanistically, deletion of XylT-I impairs the synthesis of long glycosaminoglycan chains leading to the formation of proteoglycans with shorter glycosaminoglycan chains. Histological and Second Harmonic Generation microscopy analysis revealed that deletion of XylT-I accelerated chondrocyte maturation and prevents chondrocytes columnar organization and arrangement in parallel of collagen fibers in the growth plate, suggesting that XylT-I controls chondrocyte maturation and matrix organization. Intriguingly, loss of XylT-I induced at embryonic stage E18.5 the migration of progenitor cells from the perichondrium next to the groove of Ranvier into the central part of epiphysis of E18.5 embryos. These cells characterized by higher expression of glycosaminoglycans exhibit circular organization then undergo hypertrophy and death creating a circular structure at the secondary ossification center location. Our study revealed an uncovered role of XylT-I in the synthesis of proteoglycans and provides evidence that the structure of glycosaminoglycan chains of proteoglycans controls chondrocyte maturation and matrix organization.

## Introduction

Proteoglycans (PGs) are important bio-macromolecules composed of core protein and glycosaminoglycans (GAGs) chains [1, 2]. They are involved in various biological processes such as extracellular matrix deposition, cell proliferation, adhesion, migration and differentiation [3, 4]. They play an essential role in storage of growth factors and cytokines, and in establishing a morphogen gradient during tissue development [5, 6]. PGs are involved in multiple pathological situations such as cancer, atherosclerosis, osteoarthritis and skeleton disorders [7–10] and their biological activity is highly related to their GAG chains. Biosynthesis of GAG chains is catalyzed by several glycosyltransferases and requires formation of common tetrasaccharide linker GlcA-β1-3-Gal-β1-3-Gal-β1-4-Xyl-Ser. This process is initiated by Xylosyltransferase I (XylT-I) and II (XylT-II) which catalyze the transfer of xylose from UDP-xylose to a specific serine residue of the core protein. Once the linker is formed, GAG chains are extended by addition of repeating disaccharides units forming chondroitin-sulfate (CS) and heparan-sulfate (HS) GAG chains by CS and HS polymerizing enzymes. During skeletal development, different PGs are expressed in a highly defined pattern that is regulated spatially and temporally. Versican is expressed in early stages of chondrogenesis, inversely, aggrecan is expressed during establishment and maturation of the chondrogenic cells [10]. Chondrogenesis is the earliest step for endochondral bone formation. It begins with the condensation of mesenchymal stem cells which differentiate into chondrocytes expressing type II collagen as well as cartilage specific PGs. After a period of cell proliferation, chondrocytes undergo maturation and become organized into four distinct zones i.e. resting zone, proliferative zone, prehypertrophic zone and hypertrophic zone forming a growth plate [11]. The development of the growth plate is a highly regulated process which involves several transcription factors, growth factors and extracellular matrix (ECM) compounds including PGs [11–13].

Recently, several clinical studies have reported mutations in *Xylt1* gene associated with Desbuquois Dysplasia type 2 (DBQD type II) among different ethnicities. These patients have short stature, flat face, narrow thorax and intellectual disability [14–16]. Here we generated global and chondrocyte targeted Xylt1 knockout mice and showed that XylT-I is critical for the synthesis of PGs in resting and proliferative chondrocytes and reveal that it controls the synthesis of long GAG chains of PGs. We bring evidence that XylT-I prevents hypertrophy of chondrocytes and maintains collagen fibers arrangement and chondrocyte organization in the growth plate.

## Results

### XylT-I knock-out mice display dwarfism and skeletal defects

**Fig. 1:**
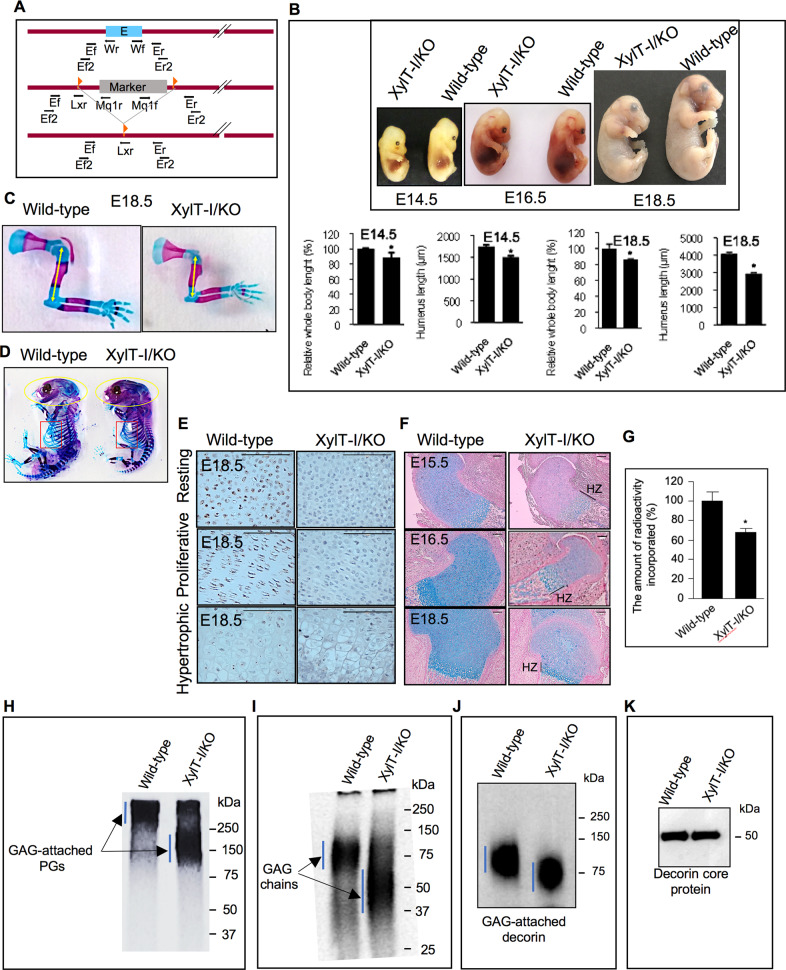
XylT-I/KO embryos display dwarfism and defects in PG synthesis. **A** XylT-I/KO embryos were generated by deletion of the Exon1 of *Xylt1* gene and a part of the promoter sequence resulting in a deletion of 18.5 kb gene sequence. Exons (E) are denoted by boxes and horizontal bar denote introns. **B** XylT-I/KO and wild-type embryos at embryonic stages E14.5, E16.5, E18.5 showing an overall reduction in body size and a frontonasal dysplasia in XylT-I/KO embryos. Graphs showing relative body and humerus length of XylT-I/KO embryos and their wild-type littermates at E14.5 and E18.5 stages, values are normalized such that control body length are set to 100%. **C** Sections through humerus at E18.5 stage stained with Alizarin red and Alcian blue (the length is represented by yellow arrow). **D** Wild-type and XylT-I/KO whole body embryos at E16.5 stained with Alizarin red and Alcian blue. XylT-I/KO embryos show frontonasal dysplasia with flat midface (yellow cercle) and skull dysplasia with narrow rib cage (red square). **E** In situ mRNA hybridization analysis of XylT-I expression assessed by RNAscope on sections of proximal humerus growth plate in XylT-I/KO and wild-type embryos at E18.5. **F** Sections of proximal humerus growth plate of wild-type and XylT-I/KO embryos at E15.5, E16.5 and E18.5 stained with Alcian blue which show strong reduction in GAGs in XylT-I/KO except in hypertrophic zone (HZ). Data are expressed as mean ± S.D. *P < 0.05, Student’s *t* test. Representative images from three independent experiments are shown (*n* = 3). Scale bar: 100 μm. **G** Synthesis level of PG-GAG chains in wild-type and XylT-I/KO primary chondrocytes analyzed by ^35^S-sulfate incorporation into GAG chains. **H** SDS-PAGE analysis of radiolabelled PGs and (**I**) GAGs chains in primary chondrocytes of wild-type and XylT-I/KO embryos. **J** Western blot detection of decorin from cultured medium of wild-type and XylT-I/KO primary chondrocytes. **K** Western blot detection of decorin from cultured medium of wild-type and XylT-I/KO primary chondrocytes following degradation of GAG-attached chains by treatment with chondroitinase ABC. Representative images from three independent experiments are shown (*n* = 3).

To elucidate the role of XylT-I in skeletogenesis, XylT-I/KO mice were generated for the first time by deletion of a part of the promoter region and of the Exon1 of the *Xylt1* gene (Fig. 1A). Embryos are collected at different stages of embryonic development. At the embryonic day E14.5, most XylT-I knock-out embryos were found at Mendelian ratio. At all stages, the size of these embryos was remarkably smaller compared to wild-type and displayed frontonasal hypoplasia with reduced air pinnae (Fig. 1B). XylT-I/KO embryos at E14.5 showed 11% reduction of the whole-body length, and 14% reduction in humerus length (Fig. 1B). At E18.5 the length of XylT-I/KO embryos was 13.5% reduced and their humerus was reduced by 28%, compared to wild-type embryos (Fig. 1B, C). Whole skeletal staining showed that mutant embryos exhibit pronounced dwarfism and apparent frontonasal hypoplasia with shorter and thinner ribs resulting in a flattened, bell-shaped thoracic cavity (Fig. 1D). At one day within the birth XylT-I/KO pups died shortly afterwards probably as a result of respiratory failure.

In situ hybridization analysis showed that XylT-I is expressed in resting, proliferative and pre-hypertrophic zones, and not expressed in hypertrophic zone in the growth plate of wild-type embryos (Fig. 1E). However, as expected, no expression of XylT-I was observed in XylT-I/KO growth plate (Fig. 1E).

### XylT-I gene deletion impairs the synthesis of long GAG chains

To determine the effect of XylT-I knock-out on PG synthesis, longitudinal sections of humerus epiphyses of XylT-I/KO and wild-type embryos at different stages of embryonic development i.e. E15.5, E16.5 and E18.5 were stained with Alcian blue. Interestingly, a strong reduction in Alcian blue staining (90%, 75% and 80%) was revealed in XylT-I/KO embryos growth plate at E15.5, E16.5 and E18.5, respectively, compared to wild-type littermates, indicating defects in synthesis and deposition of PGs in XylT-I/KO growth plate (Fig. 1F). Remarkably, whereas PG content is dramatically reduced in resting and proliferative zones in XylT-I/KO growth plate, it is less affected in the hypertrophic zone (Fig. 1F), indicating that synthesis of PGs in hypertrophic zone did not rely on XylT-I. In agreement, we showed that in contrast to resting and proliferative zones, XylT-I is not expressed in hypertrophic zone (Fig. 1E). Given that the growth plate of XylT-I/KO mice retains weak expression of GAGs in resting and proliferative zones and high expression in hypertrophic zone, it is reasonable to speculate that another xylosyltransferase may participate in the synthesis of GAGs in the growth plate. Therefore, we thought to determine whether XylT-II, the isoform of XylT-I, was expressed in the growth plate and whether deletion of XylT-I affects its expression. RNAscope analysis of mRNA expression of XylT-II showed that it is not expressed either in wild-type or XylT-I/KO embryos growth plate (data not shown), therefore suggesting the implication of unidentified xylosyltransfease.

To further investigate the effect of XylT-I deficiency on PG synthesis, PGs in XylT-I/KO and wild-type primary chondrocytes were metabolically radiolabeled by ^35^S-sulfate and the level of GAG synthesis, as determined by the amount of ^35^S-sulfate incorporation into neosynthesized GAG chains, was evaluated. The results showed that the level of GAG synthesis was decreased by 40% in XylT-I/KO primary chondrocytes, compared to wild-type (Fig. 1G). Importantly, SDS-PAGE analysis of radiolabeled PGs showed that PGs in XylT-I/KO chondrocytes were predominantly of smaller molecular size compared to wild-type (Fig. 1H), indicating that loss of XylT-I induces defects in the synthesis of PGs. Since the size of PGs depends mainly on the size of attached GAG chains, ^35^S-labeled GAG chains were released from PG core proteins by protease degradation and analyzed by SDS-PAGE. Interestingly, GAG chains of PGs produced in XylT-I/KO chondrocytes were predominantly of smaller size, compared to wild-type (Fig. 1I). To further confirm that PGs in XylT-I/KO mice contain shorter GAG chains, we performed immunoblot analysis of decorin, a PG with a chondroitin/dermatan-sulfate GAG chain secreted by chondrocytes. As shown in Fig. 1J, decorin in XylT-I/KO chondrocytes is of a lower molecular mass compared with decorin in wild-type chondrocytes. Analysis of decorin core protein following removal of GAG chains with chondroitinase showed that it is similar in size in both XylT-I/KO and wild-type chondrocytes (Fig. 1K). These data indicate that the lower mass of decorin in XylT-I/KO chondrocytes is due to the smaller size of the attached GAG chain. RT-qPCR analysis of the expression of HS (EXT1 and EXT2) and CS (Chsy1 and 3) polymerizing enzymes indicated similar expression in both wild-type and XylTI/KO chondrocytes (data not shown). Altogether, these results revealed that loss of XylT-I impairs the synthesis of larger size GAG chains leading to the formation of PGs attached with smaller GAG chains. These changes in the structure of PG-GAG chains may induce defects in ECM structure and organization and hence in cell-matrix interactions and signaling.

### Loss of XylT-I impairs chondrocyte organization and matrix deposition

**Fig. 2:**
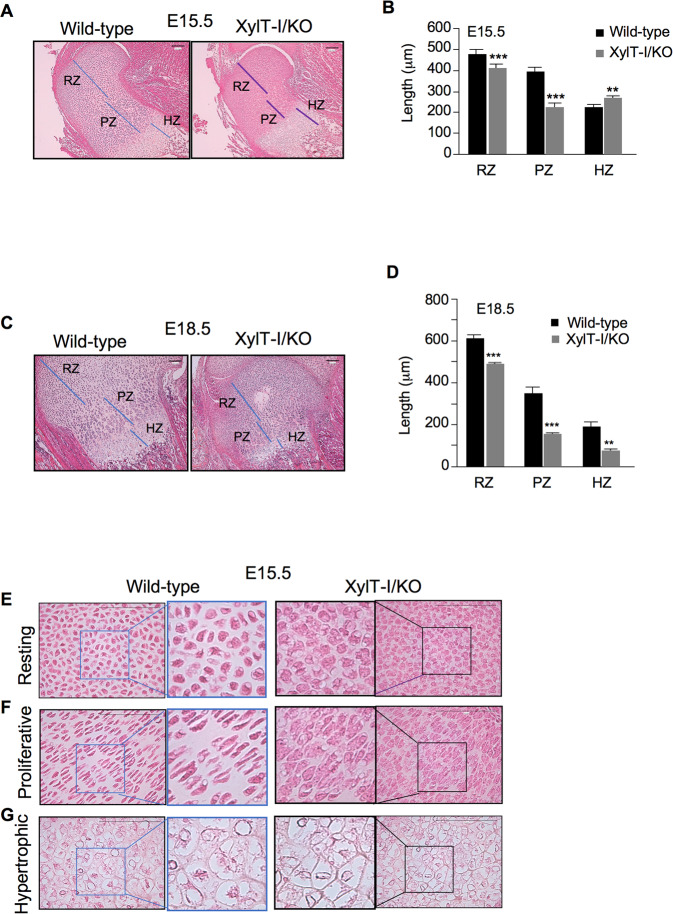
Increased hypertrophy and decreased interterritorial matrix in XylT-I mutant growth plate. **A** HES staining of sections of humerus proximal growth plate of XylT-I/KO and wild-type embryos at E15.5 showing a shortened growth plate in XylT-I/KO embryos with larger hypertrophic zone. **B** Graph showing the length of RZ, PZ and HZ in XylT-I/KO embryos and their wild-type littermates at E15.5 stage. **C** HES staining of sections of humerus proximal growth plate of XylT-I/KO and wild-type embryos at E18.5 showing a shortened growth plate in XylT-I/KO embryos. **D** Graph showing the length of RZ, PZ and HZ in XylT-I/KO embryos and their wild-type littermates at E18.5 stage. **E**–**G** Higher magnification of resting, proliferative and hypertrophic zone of the growth plate of wild-type and XylT-I/KO embryos at E15.5. RZ Resting zone, PZ Proliferative zone, HZ Hypertrophic zone. Data are expressed as mean ± S.D. *P < 0.05, **P < 0.01, ***P < 0.001, Student’s *t* test. Representative images from three independent experiments are shown (*n* = 3). Scale bar: 100 μm.

To better understand the skeletal development defect in XylT-I/KO mice, histological sections of humerus were performed and stained with HES. Compared to wild-type, the growth plate of XylT-I/KO embryos at E15.5 is shortened by about 20% and showed reduced resting (15%) and proliferative zones (45%), whereas the hypertrophic zone was larger (18%) (Fig. 2A, B), suggesting accelerated hypertrophic maturation of chondrocytes in XylT-/KO embryos. In contrast, at E18.5 the hypertrophic zone of the growth plate in XylT-I/KO embryos was shorter, compared to wild-type (Fig. 2C, D), suggesting accelerated terminal differentiation of chondrocytes and ossification at embryonic stage E18.5.

Interestingly, interterritorial matrix was severely reduced throughout the growth plate in XylT-I/KO mice compared to wild-type (Fig. 2E–G), indicating impaired deposition and/or organization of the ECM in XylT-I/KO embryos. Remarkably, while in wild-type growth plate proliferating chondrocytes were flattened and intercalate with one another to form a column (Fig. 2F), proliferating chondrocytes in XylT-I/KO growth plate were disorganized, had a rounded morphology rather that flattened shape and did not intercalate properly, as many pairs of daughter cells remained juxtaposed (Fig. 2F). This leads to an expanded growth plate which can results in a rhizomelic shortening of limb bones described in DBQD type 2 patients.

### Loss of XylT-I accelerates chondrocyte hypertrophy and ossification

**Fig. 3:**
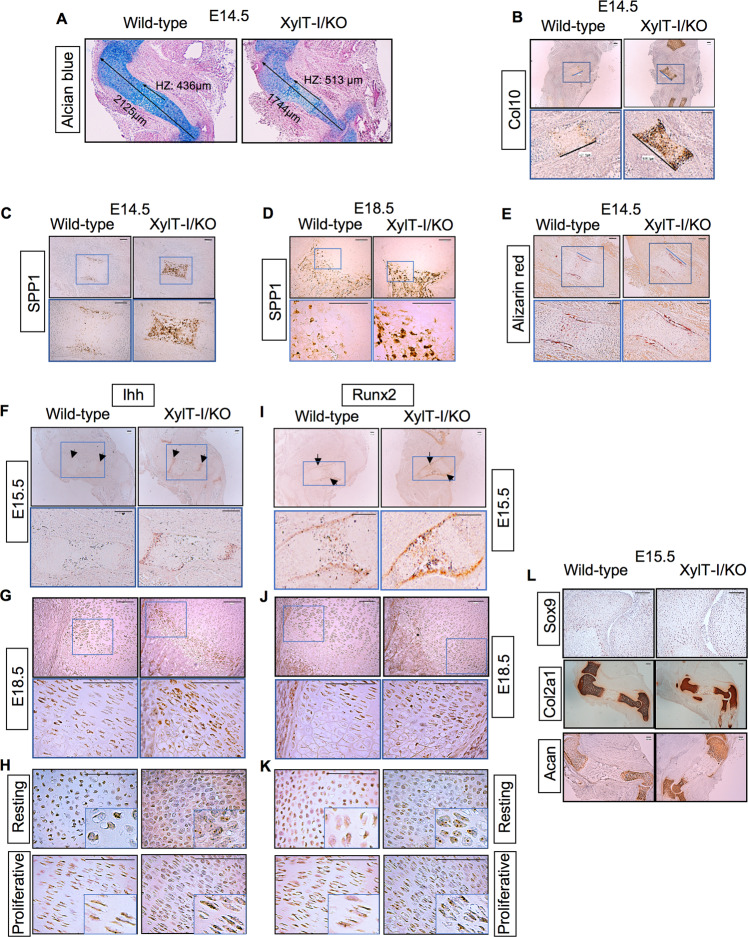
Extended hypertrophic zone and increased expression of hypertrophic and osteogenic markers in XylT-I/KO. **A** Sections through humerus of wild-type and XylT-I/KO embryos at E14.5 stained with Alcian blue (humerus and hypertrophic zone length are represented by black arrow). **B** In situ mRNA expression analysis of Col10 at E14.5 and (**C** and **D**) of SPP1 at E14.5 and E18.5 assessed by RNAscope on sections of proximal humerus growth plate in XylT-I/KO and wild-type embryos. **E** Alizarin red staining of humerus sections of wild-type and XylT-I/KO embryos at E14.5 which marks mineralized tissues. **F**–**H** In situ mRNA hybridization analysis of Ihh and (**I**–**K**) of Runx2 at E15.5 and E18.5 assessed by RNAscope on sections of proximal humerus growth plate of XylT-I/KO and wild-type embryos (**H** and **K** higher magnification of resting and proliferative zones of the growth plate of wild-type and XylT-I/KO at E18.5). **L** In situ mRNA hybridization analysis of Sox9, Col2a1 and Acan assessed by RNAscope on sections of proximal humerus growth plate of XylT-I/KO and wild-type embryos at E15.5. Representative images from three independent experiments are shown (*n* = 3). Scale bar: 100 μm.

Histological analysis of longitudinal sections of tibial epiphyses of XylT-I/KO and wild-type embryos at E14.5 showed larger hypertrophic zone in the growth plate of XylT-I/KO embryos, compared to wild-type littermates (Fig. 3A). In situ hybridization for type X collagen (Col10), a marker for hypertrophic chondrocytes confirmed the increased size of the hypertrophic zone in XylT-I/KO embryos and revealed higher expression (6.3-fold) in mutant embryos, compared to wild-type (Figs. 3B and S1A). These data suggest accelerated progression of chondrocytes towards hypertrophy that could lead to early ossification. Interestingly, analysis of the expression level of osteogenic differentiation marker osteopontin (SPP1) showed that SPP1 was highly expressed (4-fold) in the hypertrophic zone of XylT-I/KO embryos at E14.5, compared with wild-type littermates (Figs. 3C and S1A). The expression of SPP1 in XylT-I/KO embryos remains higher at E18.5, compared to wild-type (Figs. 3D and S1A). In addition, staining of humerus sections with Alizarin red revealed a large mineralized region in XylT-I/KO embryos at E14.5, compared to wild-type (Fig. 3E), indicating accelerated ossification and mineralization in XylT-I/KO embryos. It is well known that Ihh, a member of Hedgehog family expressed in pre-hypertrophic chondrocytes and early hypertrophic chondrocytes, stimulates chondrocytes proliferation and activates Runx2 expression to initiate chondrocyte hypertrophy and osteoblasts differentiation [17, 18]. Remarkably, analysis of the expression of Ihh clearly showed higher expression (4-fold) in XylT-I/KO embryos growth plate at E15.5 (Figs. 3F and S1A) as well as at E18.5 (Fig. 3G), compared to wild-type. Importantly, while Ihh expression was, as expected, present in prehypertrophic and hypertrophic chondrocytes, it is detected in resting and proliferative chondrocytes in XylT-I/KO growth plate (Fig. 3H), suggesting a prehypertrophic state in XylT-I/KO mice. Interestingly, analysis of the expression of Runx2 showed significant increase (3-fold) in XylT-I/KO embryos growth plate at E15.5, compared to wild-type (Figs. 3I and S1A), suggesting increased ossification process. Increased expression of Runx2 was maintained in hypertrophic chondrocytes in XylT-I/KO growth plate at E18.5 (Figs. 3J and S1A) and was detected in resting and proliferative chondrocytes (Fig. 3K). These results indicated that loss of XylT-I led to expression of hypertrophic markers Ihh and Runx 2 in resting and proliferative chondrocytes leading to an early onset of chondrocyte hypertrophy. Of note, analysis of the expression of Sox9 and chondrogenic markers i.e. Col2a1 and aggrecan (Acan) showed slight increase (5%, 10% and 15%, respectively) in XylT-I/KO embryos, compared to wild-type embryos (Figs. 3L and S1A). Altogether, these data suggest that XylT-I regulates chondrocytes maturation and inhibits chondrocyte hypertrophy during growth plate development.

### XylT-I gene deletion induced invasion of the epiphysial center by cells from the perichondrium

**Fig. 4:**
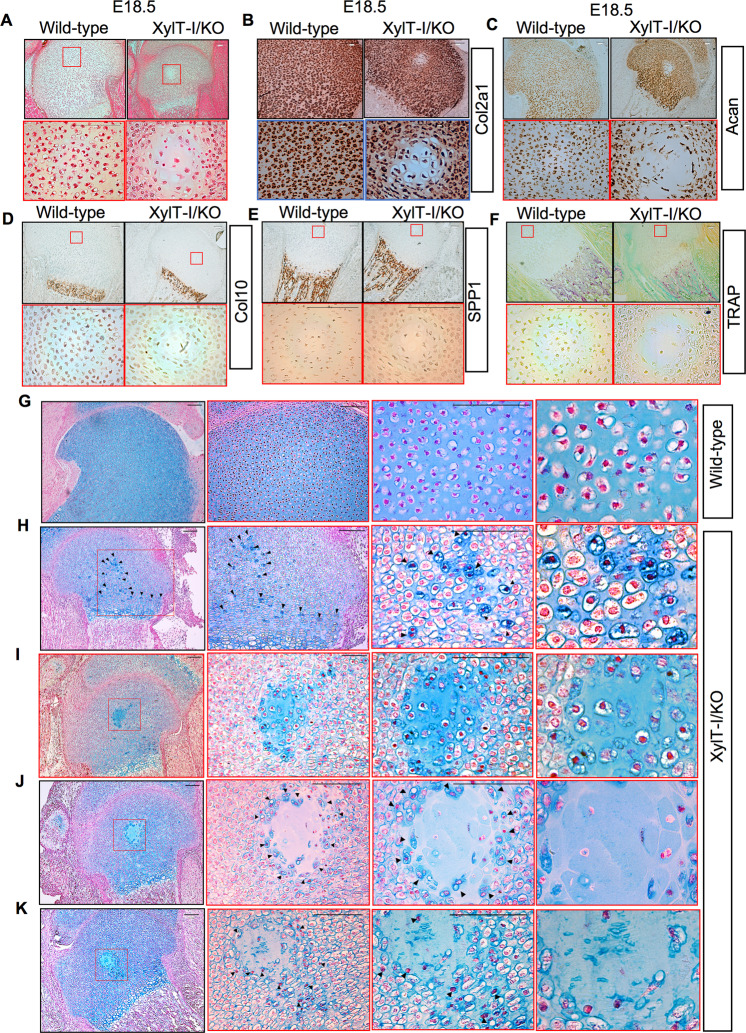
Migration of perichondrium cells to the secondary ossification center area. **A** HES staining of proximal humerus growth plate sections of wild-type and XylT-I/KO embryos at E18.5. In situ mRNA hybridization analysis of (**B**) Col2a1, (**C**) Acan, (**D**) Col10 and (**E**) SPP1 in proximal humerus growth plate sections of wild-type and XylT-I/KO embryos at E18.5 using RNAscope. **F** TRAP staining in proximal humerus growth plate sections of wild-type and XylT-I/KO embryos at E18.5. **G** Alcian blue staining of the growth plate of wild-type and (**H**) of XylT-I/KO embryos at E18.5 showing the presence in XylT-I/KO of a group of cells with condensed nuclei and strongly stained with Alcian blue migrating from the perichondrium and invading the growth plate until reaching the secondary ossification center area. **I** These cells form a circular structure and (**J**) became hypertrophic (**K**) then degraded. Representative images from three independent experiments are shown (*n* = 3). Scale bar: 100 μm.

Femoral epiphyses in mice remain uniformly cartilaginous until around postnatal day 4 (P4), with the initiation of secondary ossification center formation becoming apparent around P5-P6. Unexpectedly, histological analysis of longitudinal sections of tibial epiphyses of XylT-I/KO and wild-type embryos at E18.5 revealed the presence in XylT-I/KO embryos of hypertrophic cells within the epiphyseal cartilage region where the secondary ossification center forms, whereas no such structure was observed in wild-type embryos (Fig. 4A). This group of cells in the center of the epiphyses in XylT-I/KO is distinct from the round resting chondrocytes and is characterized by large cytoplasmic area and resemble the hypertrophic chondrocytes (Fig. 4A). mRNA in situ hybridization indicated that these cells express the chondrogenic markers Col2a1 (Fig. 4B) and Acan (Fig. 4C) but not the chondrocyte hypertrophic marker Col10 (Fig. 4D) or the osteogenic marker Spp1 (Fig. 4E). They also not stain for TRAP (Fig. 4F). Interestingly, further investigations showed the presence in XylT-I/KO embryos but not in wild-type embryos of a group of cells migrating from a region adjacent to the perichondrium next to Ranvier’s groove and invading the growth plate until reaching the secondary ossification center area (compare Fig. 4G, H). These cells showed condensed nuclei and displayed strong staining with Alcian blue (Fig. 4H). By reaching the epiphysial center, these cells exhibited a circular organization (Fig. 4I) and became highly hypertrophic in the center (Fig. 4J), before being degraded as indicated by replacement of cells by cell debris (Fig. 4K). These data showed that XylT-I deficiency triggers a yet undescribed process consisting in invasion of epiphysial center by a cell population migrating from the periphery of the growth plate adjacent to the perichondrium facing the Ranvier’s groove and leading to the formation of a circular structure in the secondary ossification center location.

### Loss of XT-I increases expression of FGFR3 and activates ERK1/2 signaling pathway

**Fig. 5:**
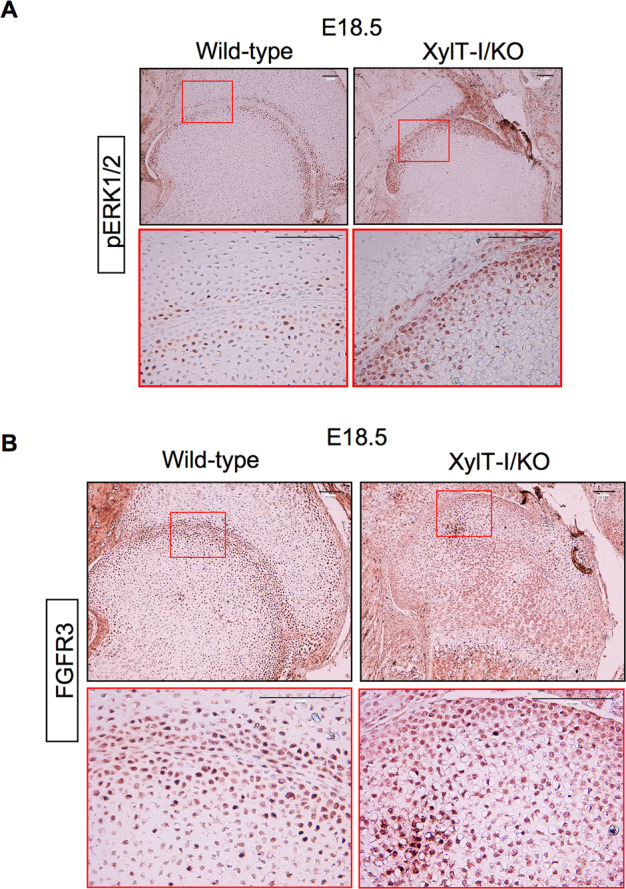
Upregulation of FGFR3 and activation of ERK1/2 in XylT-I/KO embryos. **A** Immunohistochemistry of phospho-ERK1/2 in sections of humerus of wild-type and XylT-I/KO embryos at E18.5. **B** In situ mRNA hybridization of FGFR3 in the growth plate of wild-type and XylT-I/KO embryos at E18.5 assessed by RNAscope. Representative images from three independent experiments are shown (*n* = 3). Scale bar: 100 μm.

GAG features including sulfated patterns, and the overall length of the chains seem to modulate the binding activity and signaling activation for growth factors [19, 20]. We examined the phosphorylation status of kinases that are downstream effectors of growth factor signaling such as ERK1/2. The results showed that the levels of phosphorylation of ERK1/2 was increased in periarticular chondrocytes in the growth plate of XylT-I/KO embryos at E18.5 (Figs. 5A and S1A). Given that activation of FGFR3 was associated with most common achondroplasia in humans [21–23], we examined the expression of FGFR3 in the growth plate of XylT-I/KO and wild-type embryos at E18.5. In situ mRNA hybridization analysis of the expression of FGFR3 in XylT-I/KO and wild-type growth plate revealed an increased expression (2-fold) of the receptor in XylT-I/KO growth plate, compared to wild-type (Figs. 5B and S1A). These data also indicated that the receptor is expressed in periarticular and proliferative zones but not in hypertrophic zone in both wild-type and mutant embryos.

### Early post-natal deletion of XylT-I in chondrocytes causes dwarfism and induces structural bone defects

**Fig. 6:**
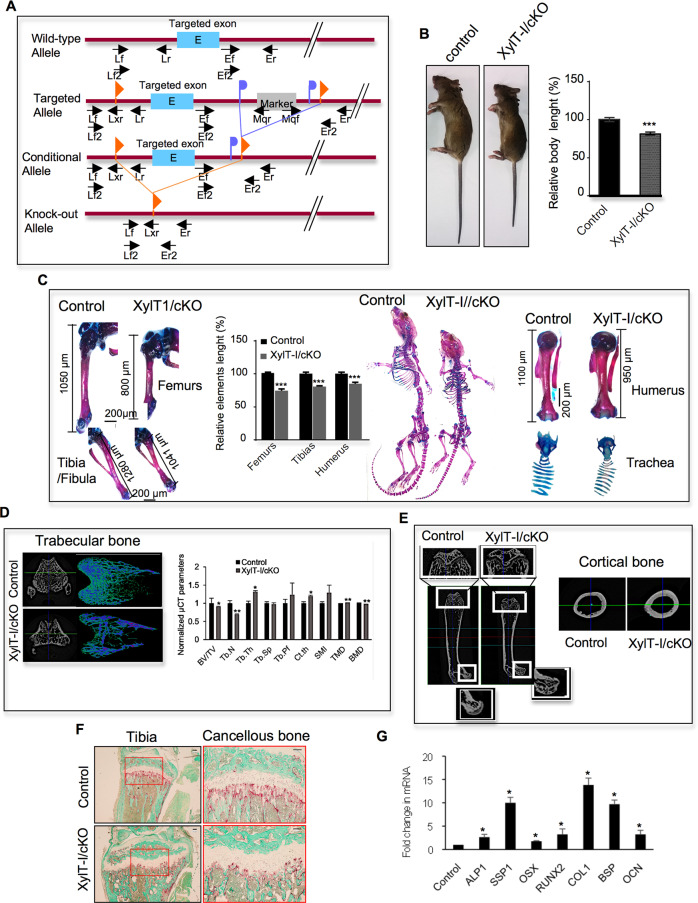
Reduced body and elements length and impaired trabecular and cortical bone formation in XylT-I/cKO mice. **A** XylT1^flox/flox^ mice with loxP sites flanking exon 5 were crossed with Col2α1-CreER^TM^ mice carrying tamoxifen-inducible Col2a1-promoter driven Cre recombinase to generate XylT1^flox/flox^; Col2α1-CreER^TM^ mice. Exon 5 is deleted following tamoxifen administration to generate XylT-I/cKO mice. **B** The body size of XylT-I/cKO mice at 8 weeks after birth shows 20% reduction in length with shortened snouts, limbs, and tails compared with control littermates. **C** XylT-I/cKO mice display reduction in femurs and tibias length. Values are normalized such that control femurs and tibias are set to 100%. Whole mount skeletal staining with Alcian blue and Alizarin red showing dwarfism phenotype characterized by shortened axial and appendicular skeletons, shortening of the skull, smaller rib cage, shortening humerus and early ossification of trachea. **D** Micro-CT scan analysis of trabecular bone in XylT-I/cKO and wild-type mice. Graph showing several measurements of trabecular and cortical bone parameters including bone volume to total volume fraction (BV/TV), trabecular number (Tb.N), trabecular thickness (Tb.Th), trabecular separation (Tb.Sp), trabecular pattern factor (Tb.Pf), cortical thickness (Ct.Th), structure model index (SMI), tissue mineral density (TMD) and trabecular bone density (BMD). Values are normalized such that control is defined as 1. **E** Micro-CT scan analysis showing larger femoral epiphysis, more developed and more ossified femoral head (white squares) and thicker cortical bone in XylT-I/cKO mice, compared with control mice. **F** TRAP staining of tibial cancellous and cortical bone in XylT-I/cKO mice, compared with control mice. **G** RT-qPCR analysis of the expression of bone formation markers and bone transcriptions factors in cortical bone in XylT-I/cKO mice, compared with control mice. Q-PCR values were normalized for the housekeeping gene ribosomal protein S29. Data are expressed as mean ± S.D. *P < 0.05, **P < 0.01, ***P < 0.001, Student’s *t* test. Representative images from three independent experiments are shown (*n* = 3). Scale bar: 100 μm.

To determine the effect of XylT-I deficiency on skeletal development in postnatal mice during growth, we conditionally removed XylT-I from cartilage by generating *Xylt1*^*flox/flox*^ mice and crossed them with *Col2α1-CreER*^*TM*^ carrying tamoxifen (TM)-inducible Col2α1-promoter driven Cre recombinase, to specifically inactivate XylT-I in a tamoxifen-inducible and chondrocyte-specific manner (Fig. 6A). Age- and sex-matched *Xylt1*^*flox/flox*^; *Col2α1-CreER*^*TM*^ mice were injected with corn oil (control) or TM (XylT-I/cKO) during the first week of age and euthanized for skeletal development evaluation at indicated age. By 8 weeks XylT-I/cKO mice were significantly smaller (20%) than control mice (Fig. 6B). Measurement of the length of long bones showed reduction of 25% and 30% in the length of femurs and tibias, respectively (Fig. 6C). Other features of the skeletal phenotype in XylT-I/cKO mice included shortening limbs with more pronounced shortening in forelimb elements, smaller ribcage, broadening of the ribs, early trachea ossification, shortening of the skull, reduced length tail and defects in cartilaginous content (Fig. 6C). Taken together, these data suggest that functional XylT-I in chondrocytes is crucial for cartilage development and endochondral bone formation during postnatal growth.

To better emphasize bone defect, structural analysis of bone was carried out using Micro Computer Tomography (µCT). µCT analysis of the trabecular bone in femurs of control and XylT-I/cKO mice demonstrated changes in trabecular bone structure, including reduced trabecular volume fraction (BV/TV), decreased trabecular number (Tb.N), increased trabecular thickness (Tb.Th) and increased tissue mineral density (TMD) in XylT-I/cKO mice, compared to wild-type mice (Fig. 6D). In addition, XylT-I/cKO mice exhibit wider femoral epiphysis and more developed femoral head than that of control mice (Fig. 6E). Interestingly, µCT analysis revealed an increase of 30% and 20% in the thickness of trabecular (Tb.Th) and cortical bone (Ct.th), respectively in femurs of XylT-I/cKO mice, compared to control (Fig. 6D, E), suggesting increased osteoblasts activity and/or impaired resorption. To test whether bone resorption and/or formation is impaired in XylT-/cKO mice, we first stained the femurs from XylT-I/cKO and control mice by TRAP (tartrate-resistant acid phosphatase) to determine whether the osteoclastic bone resorption is disturbed in mutant mice. Staining revealed TRAP-positive cells at several bone areas including the chondro-osseous junction, trabecular bone and cortical bone of XylT-I/cKO and control mice (Fig. 6F). However, chondro-osseous junction and trabecular bone areas showed a decrease in the TRAP positive osteoclast surface in XylT-I/cKO mice, compared with control mice (Fig. 6F), suggesting lower resorption in XylT-I/cKO mice. To test whether bone formation activity is increased in cortical bone of mutant mice, the expression of type I collagen (COL1), osteocalcin (OCN), osteopontin (SSP1), bone specific protein (BSP) and alkaline phosphatase (ALP1) considered as bone formation markers was analyzed by RT-qPCR in cortical bone of XylT-I/cKO and control mice. Interestingly, the results revealed that the expression of the bone formation markers was increased in cortical bone of XylT-I/cKO mice compared to control mice (Fig. 6G), suggesting enhanced osteoblasts activity in mutant mice. Importantly, analysis of the expression of Runx2 and Osx, the osteoblast transcription factors that regulate osteoblast differentiation and osteoblast bone matrix synthesis showed significant increase in cortical bone of XylT-I/cKO mice, compared to control mice (Fig. 6G). These data strongly suggest that osteoblasts in the cortical bone of XylT-I/cKO exhibit enhanced secretory activity and/or enhanced differentiation rate. Altogether, these results suggest that bone thickening in mutant mice resulted from enhanced bone formation and decreased bone resorption.

### Post-natal loss of XylT-I disturbs chondrocyte and collagen organization and accelerates bone formation

**Fig. 7:**
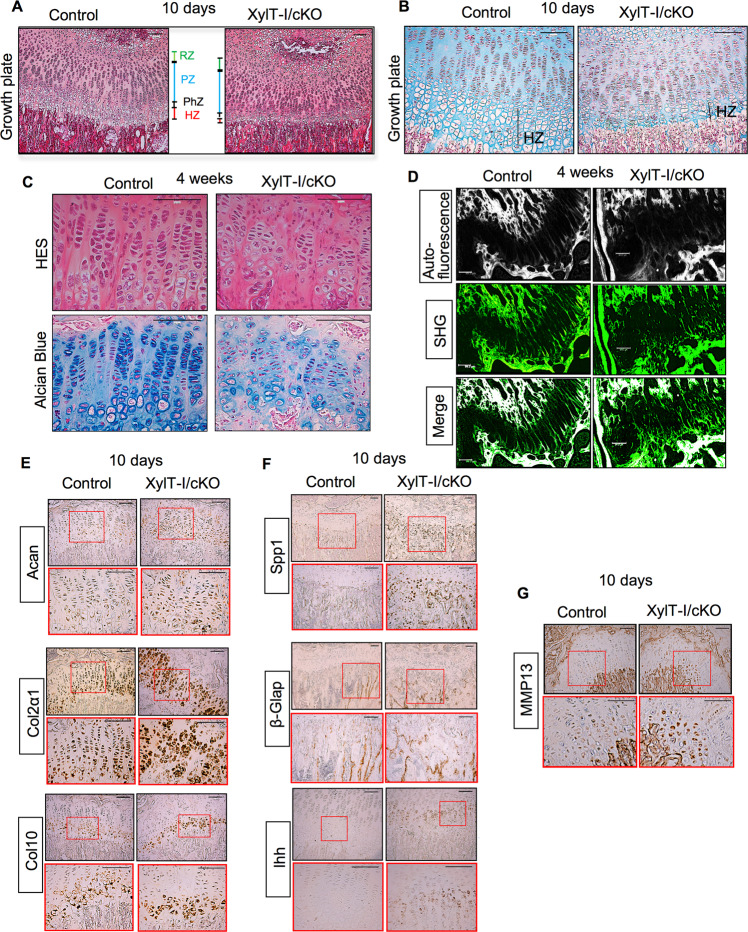
Disruption in chondrocytes and collagen fibers organization and accelerated maturation of chondrocytes in the growth plate of XylT-I/cKO mice. **A** HES staining of tibias sections of 10 days old XylT-I/cKO and control mice showing severe reduction of the hypertrophic zone in XylT-I/cKO mice. **B** Alcian blue staining of PGs revealed significantly decreased in GAG content in the resting and proliferative zones but not in hypertrophic zone in XylT-I/cKO mice, compared to control mice. **C** HES and Alcian blue staining of tibias growth plate of 4 weeks old XylT-I/cKO and control mice show disruption of chondrocytes columnar organization and reduced PGs content in XylT-I/cKO mice. **D** Second Harmonic Generation (SHG) imaging assessment of collagen fibers in the growth plate of 4 weeks old XylT-I/cKO and control mice showing collagen fibers run parallel to each other in control mice but disorganized and oriented in multiple directions in XylT-I/cKO mice. Autofluorescence (grey) and SHG signal (green) indicates the collagen in the tissue. **E** In situ mRNA expression analysis showing increased expression of the chondrogenic markers Acan and Col2α1, and of hypertrophic marker Col10 in 10 days old XylT-I/cKO mice compared to wild-type mice. **F** In situ mRNA expression analysis showing increased expression of Spp1, β-Glap and Ihh and (**G**) of the terminal hypertrophic differentiation marker MMP13 in the growth plate of 10 days old XylT-I/cKO mice, compared with control mice. RZ Resting zone, PZ Proliferative zone, PhZ Prehypertrophic zone, HZ Hypertrophic zone. Representative images from three independent experiments are shown (*n* = 3). Scale bar: 100 μm.

To explore the cellular mechanism by which XylT-I regulates skeletogenesis, knee joints from 10 days old XylT-I/cKO and control mice were examined histologically. The tibia growth plates from control mice and XylT-I/cKO mice showed that the prehypertrophic and hypertrophic zones were severely reduced in XylT-I/cKO mice compared with control mice (Fig. 7A), suggesting accelerated terminal diffentiation of chondrocytes. In addition, the staining for GAGs was severely reduced in the growth plate of XylT-I/cKO mice, compared to control except in the hypertrophic zone which showed similar PG-GAG content as in control mice (Figs. 7B and. S1B). Interestingly, histological analysis of the growth plate of 4 weeks old XylT-I/cKO and control mice showed that chondrocytes in the proliferative zone do not intercalate upon cell division and form cell clusters rather than organized column in mutant mice (Figs. 7C and S1B). Furthermore, Alcian blue staining shows the presence of only small amounts of PGs in the pericellular matrix of XylT-I/cKO mice (Fig. 7C), indicating impaired synthesis of GAGs. Altogether these results indicate that XylT-I is critical for PG synthesis and for columnar organization and maturation of chondrocytes postnatally.

The growth plate ECM plays a crucial role in the arrangement and geometry of chondrocytes, the morphology of the growth plate and the elongation of bones. It is mainly composed of collagens and PGs. The ECM located between the columns of chondrocytes (interterritorial) contains collagen fibrils that largely run parallel to each other and are proposed to participate in guiding the chondrocytes to arrange in a columnar structure [24]. To assess the organization of collagen in the growth plate of XylT-I/cKO mice, Second Harmonic Generation (SHG) microscopy allowing for high-resolution assessment of collagen fibers thickness and orientation in cartilage was used [25]. As expected, the intercolumnar collagen fibers run parallel to each other in the growth plate of control mice. In contrast, they are randomly oriented in XylT-I/cKO mice (Fig. 7D), indicating profound alterations in the organization of intercolumnar collagen fibrils in XylT-I/cKO mice growth plate. These data indicate that XylT-I supports the organization of collagen fibrils in the growth plate and suggest that loss of columnar organization of chondrocytes may result, in part, from disorganization of intercolumnar collagen fibrils network.

We showed above that prehypertrophic and hypertrophic zones were severely reduced in XylT-I/cKO mice compared with control mice, suggesting accelerated chondrocyte maturation.

To determine whether maturation was proceeding normally in XylT-I/cKO mice, we examined markers of chondrocyte maturation in the growth plate by in situ hybridization. We evaluated the expression of chondrogenic markers Acan, Col2α1 and Col10 and found that their expression is increased in XylT-I/cKO mice, compared to control (Figs. 7E and S1C). Of note, the hypertrophic region indicated by Col10 expression is noticeably reduced in XylT-I/cKO mice compared with that of control mice, thus confirming reduced hypertrophic zone in mutant mice. This may result from a higher percentage of cells exiting the hypertrophic state towards terminal differentiation and ossification. In line with this, osteopontin gene (*Spp1*) and MMP13 which are expressed in terminal hypertrophic chondrocytes and in osteoblasts were highly expressed in XylT-I/cKO mice, compared to control mice (Figs. 7F, G and S1C). As observed for osteopontin, the expression of osteocalcin gene (β-Glap) and Ihh was also higher in XylT-I/cKO mice (Figs. 7F and S1C). High expression of osteopontin and osteocalcin suggests enhanced endochondral ossification in XylT-I/cKO mice. Altogether, these results suggest increased bone formation in XylT-I/cKO mice and support the notion that the loss of XylT-I expression in chondrocytes not only affect chondrogenesis but also collagen fibers organization and bone formation.

## Discussion

Recently, studies described the characterization of zebrafish mutant line carrying point mutations in the ortholog of XylT-I and in pug mice with hypomorphic allele of XylT-I [26, 27]. Here, we generated global and chondrocyte targeted knock-out mice of *Xylt1* and showed that XylT-I/KO embryos and XylT-I/cKO mice in which XylT-I was deleted postnatally and specifically in differentiated chondrocytes exhibit dwarfism phenotype as observed for XylT-I/KO embryos, suggesting that XylT-I plays a critical role in chondrocyte maturation rather than in chondrocyte differentiation. Loss of XylT-I in cartilage leads to dramatic reduction in PG-GAG content in resting and proliferative zones but not in hypertrophic zone, suggesting that XylT-I drives the synthesis of PG-GAGs in early but not late stages of chondrocyte maturation. Accordingly, XylT-I was not detected in hypertrophic chondrocytes and knock-out of XylT-I led to hypertrophy-like phenotype of resting and proliferative chondrocytes, suggesting that XylT-I prevent hypertrophy of chondrocytes. Of note, it has been shown that the expression of XylT-I is significantly increased in early chondrogenic maturation [28]. Mechanistically, loss of XylT-I impairs the synthesis of larger sized GAG chains in chondrocytes leading to the formation of PGs with shorter GAG chains. Of note, the expression of HS and CS polymerizing enzyme in XylT-I/KO and wild-type chondrocytes indicated similar levels of expression. How XylT-I controls the synthesis of long GAG chains despite the fact that elongation of the chains, which is a non-template process, is mediated by the activity of GAG polymerizing enzymes is unclear. Nevertheless, we can hypothesize that PGs with GAG chains initiated by XylT-I may be delayed in secretion and/or sorted differently in the Golgi. Ongoing studies will hopefully lead to identify the mechanism involved.

Long-chain GAG polymers can string together multiple proteins acting as a concatenator, bringing multiple receptor/ligand complexes together. Therefore, loss of long-GAG chains may lead to structural changes in ECM and to defects in matrix-cell interactions and signaling. Indeed, XylT-I mutant mice growth plate display hypertrophic-like chondrocytes, reduced interterritorial matrix, disorganized columnar chondrocytes and collagen fibers. Of note, collagen fibers organization is suggested to play an important role in constraining chondrocytes to arrange in a columnar structure which is important for bone longitudinal growth [24, 29, 30]. Although we have not addressed the mechanism underlying loss of collagen organization, reduced length of PG-GAG chains following XylT-I deletion may probably unable PGs such as decorin to fulfil the function of normal PGs regarding collagen fibrillogenesis and assembly of fibrils into mature matrix suprastructures [31–33].

Unexpectedly, the growth plate of XylT-I/KO embryos at E18.5 showed the presence in epiphyseal center of a structure that resembles to the secondary ossification center. An important event in secondary ossification center formation is the appearance of cartilage canals at postnatal, which bring vascularization to the epiphyseal region and, eventually, invading osteogenic cells [34]. However, no such structure has been observed in XylT-I/KO embryos. In contrast, cells in the epiphyseal center became hypertrophic and express Col2a and aggrecan and were negative for TRAP staining, suggesting that they rise from chondroprogenitors. Interestingly, these cells did not seem to originate from within the resting zone, but rather migrated initially from the periphery of the growth plate, immediately adjacent to the perichondrium facing the Ranvier’s groove. Ranvier’s groove contains two type of skeletal progenitors i.e. osteoprogenitors that differentiate in osteoblasts and undifferentiated mesenchymal cells presumably chondroprogenitors [35, 36]. Previous studies have shown the migration of cells from perichondrium facing the groove of Ranvier to resting zone but they either clonally give rise to a full column of chondrocytes [37] or provide a source of resting zone chondrocytes as the growth plate expands transversely [38]. As the embryos died after birth, we unfortunately can’t follow the development of this structure.

Several skeletal dysplasias are associated with gain-of-function mutations in FGFR3 [39]. Interestingly, we found that expression of FGFR3 is increased in the growth plate of XylT-I/KO embryos associated with increased phosphorylation of ERK1/2. However, activation of ERK1/2 was observed mainly in periarticular zone, whereas that of FGFR3 was prominent in proliferative zone and to lesser extent in periarticular zone. These data suggest that increased phosphorylation of ERK1/2 in periarticular zone is not due to FGFR3. On the other hand, XylT-I/KO embryos and XylT-I/cKO mice showed an increased expression of Ihh and Runx2. It is well known that Ihh signaling plays a major role in chondrocyte maturation and stimulates Runx2 expression in the perichondrium [17, 18, 40–42]. Upregulation of Runx2 in XylT-I/KO growth plate may lead to early chondrocyte hypertrophy and ossification. Accordingly, the expression of SPP1 was detected in XylT-I/KO growth plate at E14.5, but not in wild-type embryos indicating an early ossification process in mutant embryos. Noteworthy, Ihh acts also on the perichondrium to stimulate formation of the bone collar by promoting osteoblast differentiation of the perichondral progenitor cells [43]. We found that the expression of the bone formation markers was increased in cortical bone of XylT-I/cKO mice, compared with control. In addition, bone resorption was decreased in XylT-I/cKO mice, therefore suggesting that bone thickening in mutant mice resulted from enhanced bone formation and decreased bone resorption.

## Materials and methods

### Mouse strains and genotyping

The animal experiments were conducted according to the recommendations of European Directive 2010/63/UE and French legislation article R.214-88. The study was approved by Ministere de l’Education Nationale (APAFIS#10024). During acclimatization and experiments, the animals were kept in standard conditions of temperature (23 ± 2°C) and light-controlled environment (12 h light/12 h dark cycle), and with free access to water and pelleted food. XylT-I/KO mice were generated by deletion of 10.5 kb (promoter and exon1) of the *Xylt1* gene. Genotyping was performed by PCR using specific primers i.e. Ef: 5’CTCATTCCATGGTGAACA CGGG3’, Wr: 3’GCTCTTCATTCATTCACATGTCCTCAT CACC5’, Ef2: 5’ACAGAATTTGCAG CATATCAACATGATC3’ and Lxr: 3’GAAGTTATA CTAGCGGCCGTTCAC 5’. XylT-I/KO homozygous yielded DNA bands at 364 pb with Ef2/Lxr primers. To generate the *XylT1*^*flox/flox*^; *Col2α1-CreER*^*TM*^ mice, transgenics *Col2α1-CreER*^*TM*^ mice (BALB/c background) were crossed with *XylT1*^*flox/flox*^ mice (C75BL/6 background). Offspring were genotyped by PCR for *Col2α1-CreER*^*TM*^ and *XylT1*^*flox/flox*^ using specific primers i.e. Lf (5’ATCCCTAAGATGTGTTTTCCAGTCA CCAT3’), Lr (5’GAGTTA GTTAACCAGTGGGCTTGAGGTG3’), Ef (5’CTAGAGATGACTGACTGGCCCTGGG A3’), Er (5’TTTCCAAGGCAGCACCTCTAGTTCA3’) and Lxr (5’CGAAGTTA TCTGCAG GTCGACCTTAAG3’). *XylT1*^*flox/flox*^; *Col2α1-CreER*^*TM*^ homozygous yielded DNA bands at 300 pb with Lf/Lr primers, 427 pb with Ef/Er primers, 209 pb with Lf/Lxr primers and 100 pb with Cre primers. The PCR cycling parameters were 35 cycles of denaturation at 94 °C for 30 s, annealing 55 °C for *Cre* and 62 °C for *XylT1* for 30 s, and elongation for 60 s at 72°C. XylT-I/cKO and control mice were obtained following intraperitoneal injection with tamoxifen 100 µg/g body weight or with corn oil (carrier), during the first week of age. Mice were then euthanized at 10 days, 4 or 8 weeks old.

### Skeletal staining

Embryos and mice were dissected and fixed overnight in 96% ethanol. Cartilage elements of skeletons were stained in 0.03% Alcian blue dye (0.03 g Alcian blue (8GX, Sigma) dissolved in 80 ml 95% ethanol and 20 ml glacial acetic acid). Skeletons were washed twice with 95% ethanol and then placed in 1.5% KOH until the remaining soft tissues were dissolved. Bones/mineralized tissues were stained in 2% Alizarin red (100 ml 1% KOH, 0.02% Alizarin red (Sigma)) for 4 h and cleared in 0.8% KOH in 20% glycerol solution for 24 h, then 0.5% KOH glycerol solution for 48 h. Finally, embryonic skeletons were stored in a 50% glycerol, 50% ethanol solution.

### Histological and immunohistochemical analyses

Bone specimens were fixed in 4% formalin for 24 h at room temperature, decalcified and dehydrated. The sample was embedded in paraffin, and sections 5 μm thick were prepared. The sections were stained with either Alcian blue (0.001%)/Kernechtrot (0.001%), Safranin-O (0.1%)/Fast Green (0.03%) or Harrys’s Hematoxylin/Eosin (0.01%)/Safran (0.01%) using a Leica ASP300S autostainer (Leica, Germany). Tartrate staining was carried out using Acid phosphatase, Leukocyte (TRAP) kit (Sigma-Aldrich). Images were obtained by using Leica DMD 108 microscope (Leica).

Immunohistochemical analysis was performed using the Dako Envision kit (Agilent, CA, USA) according to the manufacturer’s instructions. Briefly, paraffin-embedded sections were dewaxed in alcohol baths, then washed by PBS, followed by incubation in citrate solution 10 mM (pH = 6) overnight at 55 °C. Subsequently, sections were blocked in BSA solution (2% w/v), incubated with rabbit anti-phospho-p44/42 MAPK (Cell Signaling Technology Cat# 4370, RRID: AB_2315112) primary antibodies for 60 min at room temperature then incubated for 30 min with the anti-rabbit HRP linked (Cell Signaling Technology Cat# 7074, RRID: AB_2099233) secondary antibody and revealed with DAB (3,3’-Diaminobenzidine). After counterstaining with Hematoxylin, the slides are mounted and digitized using a light microscope (DMD108 Leica). The staining area percentages of Alcian blue and IHC markers were determined using image analysis software ImageJ as previously described [44].

### RNAscope in situ hybridization

The RNAscope assay was performed on 5 μm formalin-fixed, paraffin-embedded tissue sections using the 2.5 HD-Brown kit (Advanced Cell Diagnostics; ACD) according to the manufacturer’s instructions. Tissue sections derived from comparable long bone locations were processed and developed simultaneously. The slides were hybridized with specific probes for XylT-I, XylT-II, Col2a1, Col10a1, Sox9, Acan, Runx2, Ihh, β-Glap, FGFR3, MMP13 and Spp1 purchased from ACD. RNA hybridization images were obtained by using Leica DMD 108 microscopy.

### Primary chondrocytes culture and metabolic labeling of GAG chains

The primary chondrocytes were prepared from embryonic ribs of XylT-I/KO and wild-type embryos according to the method described by Gosset et al. [45]. Metabolic labeling of GAG chains of PGs was carried out using [^35^S]-sulfate incorporation method as described by De Vries et al., [46]. Briefly, primary chondrocytes cells grown in 6-well culture plate were radiolabeled with 10 µCi/ml of [^35^S]-sulfate (Perkin Elmer, Courtabœuf, France) overnight. Then, conditioned culture medium was collected, digested with papain (1 mg/ml) (for GAG chains) or not (for PGs) and [^35^S]-labeled GAGs or radiolabeled PGs were precipitated by cetylpyridinium chloride (CPC) as described by Bronson et al. [47]. The CPC precipitated radiolabeled PGs and GAGs were separated by SDS-PAGE on a 4-20% Tris/Glycine gel. The gel was dried and exposed to autoradiography film.

### Western blot analysis

For decorin analysis, PGs from conditioned medium of chondrocytes cultured in 6-well plate were precipitated with CPC method and digested or not with chondroitinase ABC. The concentration was determined by Bradford method [48] and 20 μg of proteins were analyzed by Western blot using anti-decorin antibodies (R&D Systems, Cat# AF1060, RRID: AB_2090386). The protein bands were visualized by chemiluminescence using Clarity Western ECL substrate (Bio-Rad) and anti-goat HRP liked secondary antibodies. Full and uncropped western blots are presented in Supplementary File.

### RNA extraction and real time PCR

Total RNAs was isolated from the chondrocytes using RNeasy plus mini kit® (Qiagen, Germany) according to manufacturer’s instructions. RNA isolation from bone pieces was performed using TRIzol (Invitrogen, CA) and RNeasy kit (Qiagen, Germany). 500 ng of total RNA were mixed with 4 μl of SuperMix (iScript™ Reverse transcription supermix for RT-QPCR) (Bio-Rad) and supplemented with RNAse free water for a final volume of 20 μl. The mixture is incubated at 25 °C for 5 min and then at 46 °C for 20 min and finally at 95 °C for 1 min. The synthesized cDNAs are used directly for PCR or stored at −20 °C. Quantitative PCR was carried out using iTaq™ Universal SYBR Green Supermix kit (Bio-Rad) and validated RT-PCR primers for mice EXT1, EXT2, CHSY1, CHSY3, Col1, OCN, ALP1, SSP1, OSX, BSP and Runx2 (Qiagen). Cycling parameters were 30 s at 95 °C; 40 cycles of 15 s at 95 °C and 1 min à 60 °C. Gene expression was determined in triplicate in three separate experiments and normalized using the housekeeping genes ribosomal protein S29. Analyses and fold differences between the wild-type and XylT-I/cKO mice were determined with the formula 2^-ΔΔCT^. The data are relative to the control values. The expression change was calculated from the values of ΔΔCT.

### High-Resolution Micro Computer Tomography assessment (µCT)

Femurs from 8 weeks old XylT-I/cKO and control mice (*n* = 6) were scanned using SKYSCAN 1272 High-Resolution X-Ray Microtomograph (Bruker, Germany). Data were acquired at 70 keV, with 7 µm cubic resolution. Three-dimensional reconstructions were generated with Microscan 1272 Brucker.

### Second harmonic generation microscopy (SHG)

The second harmonic generation signal measurements were performed on 10 μm tibias sections of XylT-I/cKO and control mice using an APE picoEmerald laser that delivered synchronized 7 ps pulses at 800 nm to a scanning microscope (SP8-CARS, Leica Microsystems, Mannheim, Germany). In the SHG operation mode the signal was filtered to detect collagen at 400 nm in the backward direction with a hybrid detector using a large dynamic range combined with low dark noise [49]. The two-photon excited collected with hybrid detector in the forward direction and autofluorescence (grey) is used to highlight the cells, whereas the SHG signal is utilized to indicate the global collagen in the tissue. The objective lens used was a HCX PL APO 40×1.40 (water immersion). The excitation wavelength was 1064 nm and emission bandwidth was 495-610 nm for autofluorescence.

### Data analysis and statistical procedures

Data were collected from at least three mice of each genotype (*n* = 3) from independent litters and presented as average with standard error of the mean. Statistical analysis was performed using GraphPad PRISM and Student *t* test was used to determine the level of statistical significance. The significance threshold was at *P < 0.05.

## Supplementary information

Supplementary Figure 1

Original Data File

AJ-checklist

## Data Availability

All data needed to evaluate the conclusions in the paper are present in the paper. Additional data related to this paper may be requested from the corresponding author.
