# Supplementary figures and images for: Xylosyltransferase I mediates the synthesis of proteoglycans with long glycosaminoglycan chains and controls chondrocyte hypertrophy and collagen fibers organization of in the growth plate
Source: Cell Death Dis. 2023 Jun 9;14(6):355. doi: 10.1038/s41419-023-05875-0 (PMC10256685; doi:10.1038/s41419-023-05875-0)

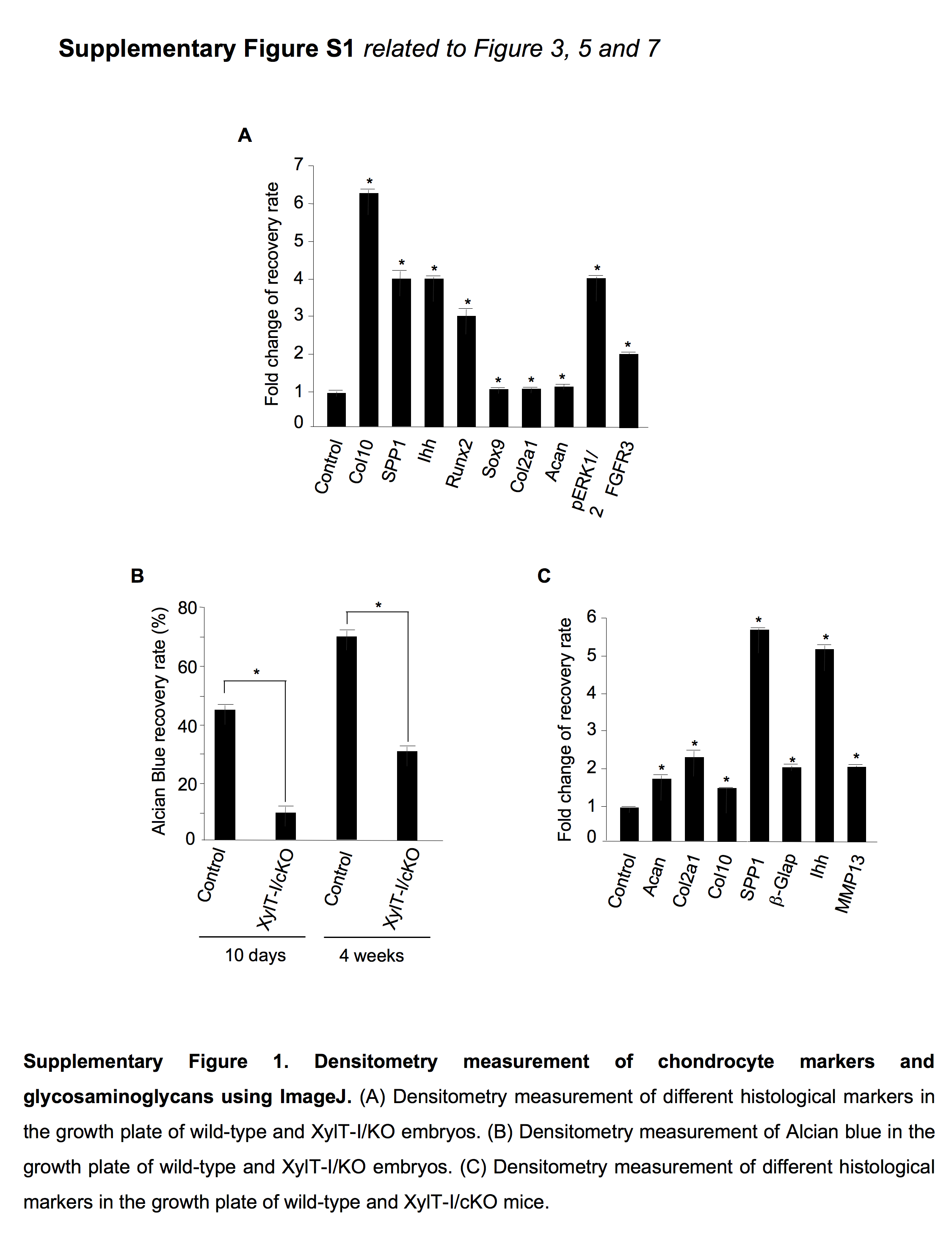

Supplement: Supplementary file 1 — Supplementary Figure 1 [file 41419_2023_5875_MOESM1_ESM.tif]

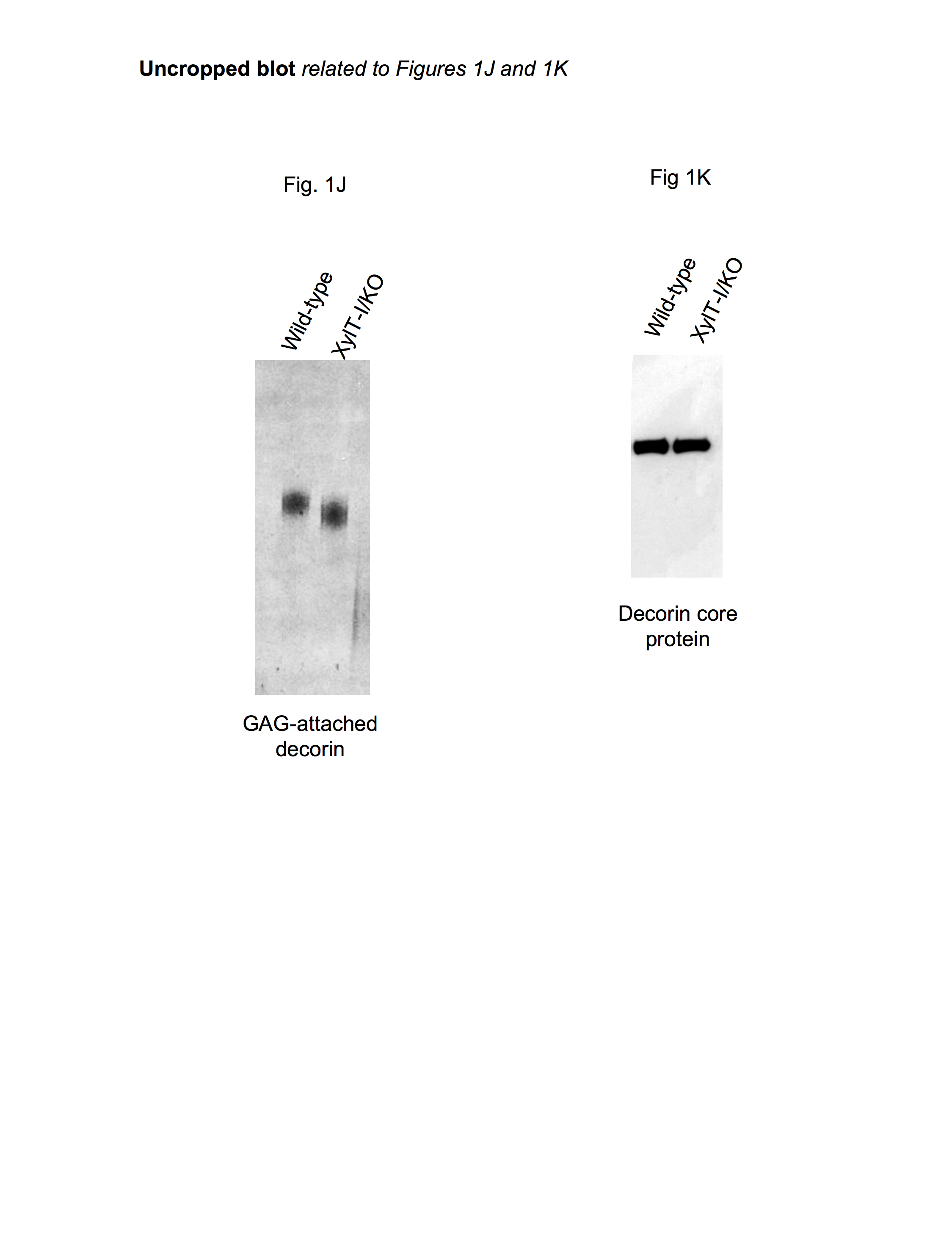

Supplement: Supplementary file 2 — Original Data File [file 41419_2023_5875_MOESM2_ESM.tif]
